# First Aid Education for Opioid Overdose Poisoning: Scoping Review

**DOI:** 10.7759/cureus.12454

**Published:** 2021-01-03

**Authors:** Jeffrey L Pellegrino, Jamillee L Krob, Aaron Orkin

**Affiliations:** 1 Emergency Management & Homeland Security, University of Akron, Akron, USA; 2 Health Sciences, Aultman College of Nursing & Health Sciences, Canton, USA; 3 Family and Community Medicine, University of Toronto, Toronto, CAN

**Keywords:** naloxone, opioid, education, review, survival, quality, first aid, responder

## Abstract

Effective health education needs ongoing evidence to support policy development and action in a public health crisis, like the opioid epidemic in the United States. Opioid Education and Naloxone Distribution (OEND) programs work to change behaviors through information, education, and resources to empower people to prevent and respond to opioid overdose poisonings. In this review, we sought to identify the first aid educational components of OEND to address opioid overdose poisoning, identify gaps in the existing literature, and support the development of future studies that could then be systematically reviewed.

From a systematic review that identified 2057 peer-reviewed manuscripts, 59 studies demonstrated that the educational literature is sparse, of low quality, lacks quality measures and effective methodologies, and suffers from self-reported and highly inconsistent endpoints, making outcome comparisons challenging, if not impossible. The reviewed OEND programs generally used a public health/health education approach focusing on people who inject opioids, their family and friends, first responders, and rarely the general public. Depending on the learners, interventions were broken down to those <15, 16-90, and >90 minutes, which categorically showed differences in knowledge and first aid response actions. Only eight studies used comparison groups which provide a slightly higher level of evidence. Reports of survival appeared to positively correlate with naloxone kit distribution. Opportunity exists to develop policies and plans that support individual and community efforts through evidence-based guidelines, particularly to the domains of first aid education, so that educators and organizations can deliver efficacious programming that meets the needs of their learners.

## Introduction and background

Poisoning from opioid overdoses is a public health epidemic in the United States (US), where opioids are implicated in almost 70% of all drug overdose deaths [1]. Moreover, each death represents many more non-fatal opioid-involved overdose poisonings that add to the social, health, and economic costs of the overall opioid epidemic [2]. Increased rates of opioid use, abuse, and overdose have been reported across the globe [3,4], which raises a humanitarian concern for the political neutrality of first aid education, resource capacities, and public health educational interventions developed to mitigate the costs to the medical system, individuals, and society.

These interventions include the use of an opioid antagonist, such as naloxone, to counter the effects of opioid overdose [5-7]. The clinical use of naloxone in opioid overdose poisonings is well established, as is the effectiveness of overdose education and naloxone distribution (OEND) programs for reducing opioid-related deaths in clinical and public health perspectives [6,8-10]. Educational competencies and local implementation outcomes for effective OEND have not been systematically examined and remain an area of uncertainty in the development and implementation of optimal OEND programs [11,12].

In 2015, the International Liaison Committee on Resuscitation’s (ILCOR) Advanced Life Support (ALS) taskforce strongly recommended the use of naloxone for individuals in opioid-associated cardiac arrest from opioid toxicity; however, at the time, the recommendation was based on low-quality evidence [13]. Also in 2015, the Basic Life Support (BLS) Taskforce of ILCOR did not make a treatment recommendation for using naloxone within resuscitation guidelines for suspected opioid overdose poisoning. Nevertheless, they did suggest offering opioid overdose response education, with or without naloxone distribution, to persons at risk for opioid overdose in any setting [14]. Today more intervention strategies exist, including training on how to recognize overdose emergencies and administer naloxone, as a first aid practice for those who are more likely to witness overdose including peers and family members of people who inject opioids (PWIO) [5,12,15]. The Education, Implementation, and Teams (EIT) taskforce of ILCOR chose to identify the educational scope of current OEND that reported outcomes. Our goal was to identify the first aid educational components of OEND to address opioid overdose poisoning, identify gaps in the existing literature, and to support the development of future studies that could then be systematically reviewed.

## Review

Methods

Our scoping review search strategy followed the PICOST (Population, Intervention, Comparator, Outcome, Study Designs and Timeframe) framework and included the following as the query scope:

Population: First aid providers, in a non-clinical environment for practice, responding to potential opioid overdose poisoning.

Intervention: Education on response/care of an individual with an opioid-associated emergency

Comparators: Another or non-specialized first aid education.

Outcomes: Any clinical or educational outcome, including survival, naloxone administration, other first aid provided, skills, attitude, knowledge.

Study Designs: Randomized controlled trials (RCTs) and non-randomized studies (non-randomized controlled trials, interrupted time series, controlled before-and-after studies, cohort studies) were eligible for inclusion. 

Timeframe: All years and all languages were included as long as there was an English abstract; unpublished studies (e.g., conference abstracts, trial protocols) were excluded. The literature search was dated to November 13, 2019. 

Inclusion and Exclusion Criteria: Manuscripts needed to report educational, first aid, clinical or population outcomes from a described educational intervention. Exclusion criteria included studies that did not meet PICOST, unpublished studies, and studies only published in abstract form, unless accepted for publication. 

**Figure 1:**
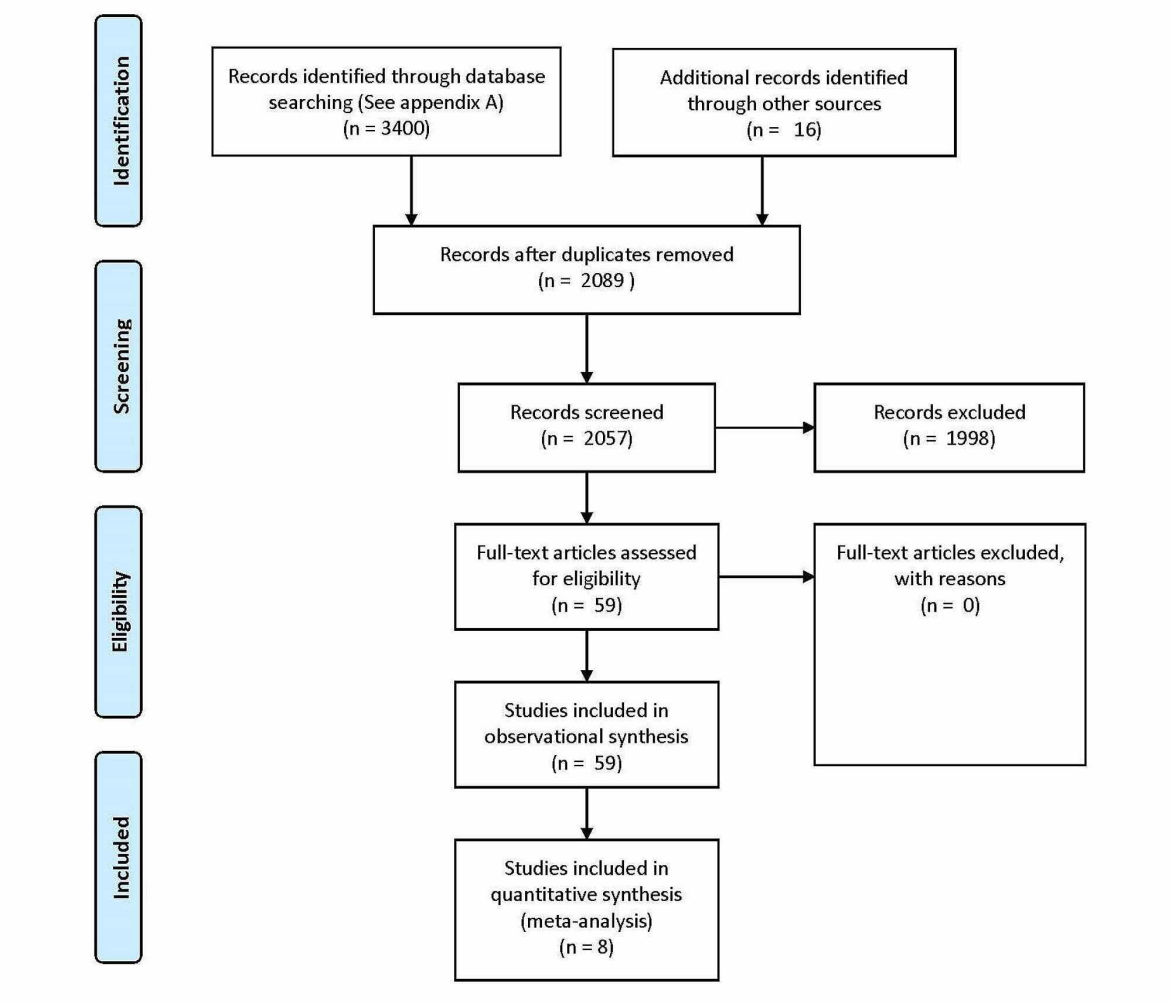
PRISMA (Preferred Reporting Items for Systematic Reviews and Meta-Analyses) Flow Diagram

The initial search yielded 3400 articles as shown in the Preferred Reporting Items for Systematic Reviews and Meta-Analyses (PRISMA) flow diagram (Figure 1). After deletion of duplicate papers, 2057 total unique articles remained. Table 1 includes a summary of the results of the database searches. Two independent reviewers (JLP and JK) screened the title and abstract of each article and in cases of discrepancy, a third reviewer (AO) was enlisted. From a systematic search of 2057, plus 16 hand-searched citations, 59 primary studies met the inclusion criteria and were reviewed in their entirety.

**Table 1:** Final Results of the Database Searches (Completed 2019-11-14)

Database	Hits
MEDLINE (Ovid)	868
Embase (Ovid)	1667
Cochrane CENTRAL Register of Controlled Trials (Ovid)	107
Cochrane Database of Systematic Reviews (Ovid)	24
Database of Abstracts of Reviews of Effects (Ovid)	3
ACP Journal Club (Ovid)	1
Cochrane Methodology Register Database (Ovid)	0
Health Technology Assessment Database (Ovid)	1
National Health Service Economic Evaluation Database (Ovid)	1
CINAHL (EBSCO)	721
ERIC (EBSCO)	7
Total citations	3400
Duplicates	1343
Total unique database citations	2057

Results

We found an insufficient number of high-quality studies to support a more specific systematic review comparing one educational intervention versus another or no educational intervention at all. As a caveat, the majority of these manuscripts were one-group pretest-posttest design. Campbell and Stanley [16] call this a “pre-experimental design” often used to find variables that need to be controlled in later experimental designs. It is not possible to draw any conclusion about effectiveness from such a design, nor extract data for comparison between studies.

Public health professionals provided training in 69% of the studies, with another 24% being facilitated by clinically trained professionals. From the 59 studies, the focused audience appeared to be PWIO (62.7%). Educational outcomes were reported by 81.4% of the manuscripts. Less than half of the manuscripts reported any first aid outcomes (47.5%).

Comparing studies with no skill practice (n=43) to those with skill practice (n=16), 84% reported improved learning outcomes to 75% respectively. This reversed with clinical outcomes, where 90% of studies reporting skill practice also reported improved results versus 79%. Therefore, on the basis of the literature reviewed, face validity does not appear to be a constant in a positive relationship between skill practice and OEND training outcomes. The review also revealed all clinical outcomes were self-reported, generally when learners came to refill their prescription for naloxone; the verifiability of this data was not reported.

Regarding length of education intervention, six of the 59 studies reported training time of ≤15 minutes (brief), all of which reported either improved educational and/or clinical outcomes. Of the 22 manuscripts reporting training time from 16-60 minutes (standalone programs) and reporting educational outcomes, 16 (73%) were improved. Of the 16 reporting clinical outcomes, 14 (88%) were improved. Of the seven education programs reporting training time >60 minutes (opioid education embedded in other prevention) and reporting educational outcomes, six (86%) were improved; of the three that reported clinical outcomes, one (33%) was improved. Therefore, on the basis of the literature reviewed, there does not appear to be a consistent relationship between OEND training duration and educational outcomes.

Contrary to expectation, interventions with no skill practice, compared to those with practice, showed inverse educational outcomes to clinical outcomes. Of those with no skill practice (n=43, 84%) reported positive learning outcomes compared to 75% with skill practice. And, clinical outcomes were positive in 90% of studies reporting skill practice versus 79% without skill practice. This may be due to the relatively low numbers of studies or participants who reported clinical outcomes.

Surveying the 59 studies through the five domains of first aid education (Prevent & Prepare, Early Recognition, First Aid/Access Help, Advance Care-Self Care) [17], the proportional differences give perspective of where educational priorities are currently and where it might be augmented in the future (Table 2). Of the 59 studies, only eight used a comparator group [18-25]. These manuscripts were charted and organized into an extraction table (see Table 3). 

**Table 2:** Survey of Manuscript Outcomes by Domain of First Aid Education (n=59) Opioid Education & Naloxone Distribution (OEND) People Who Inject Opioids (PWIO)

Survival Behavior Domain	Frequency of citations	Key Concepts
Prevent & Prepare	45 (76%)	Address impact on behaviors for carrying naloxone, storing, and any stigma. Several studies applied modified attitude assessment tools, which are not necessarily validated in the population of learners [25].
Early Recognition	52 (88%)	Some programs tailored these lessons to learners’ experience with overdoses and used different media or common language to improve knowledge and context for the need to act to save a life.
First aid / Access Help	First Aid 59 (100%) Accessing Help 42 (71%)	First aid education varied from passive, to demonstrated, to practiced skills. Contextual issues of safety from a variety of risks were often included and went beyond simple naloxone knowledge and skills in most instances. Although early activation of emergency services can improve clinical outcomes in opioid-related emergencies, past fears and adverse experiences with law enforcement personnel, and the variable protection of Good Samaritan legislation, seems to present a barrier to activation. This problem was directly addressed in multiple studies and represents a unique challenge for first aid education in this domain.
Advanced Care	8 (14%)	Opioid Education & Naloxone Distribution (OEND) programs do not necessarily address Advanced Care domain practice, but the awareness of health professionals to help those who have overdosed obtain training and naloxone, as well as recovery help, was addressed in studies, mainly within academic pharmacy and medical education programs.
Self-recovery	10 (17%)	Generally, this manifested in trying to build relationships between tertiary prevention programs and people who came to refill naloxone prescriptions. Some programs dealt with mental trauma for lay responders in light of social push back from the people who inject opioids (PWIO) or peers, who may perceive an injustice in taking away a “high.”

**Table 3:** Extracted manuscripts with comparison intervention (n=8) Opioid Education & Naloxone (OEND) Opioid Overdose Knowledge Scale (OOKS) Opioid Overdose Attitudes Scale (OOAS) Pre-Intervention Group Without Training in Overdose Prevention (PREIGW) Pre-Intervention Group with Sporadic Training in Overdose Prevention (PREIGS) Comparison Group (CG) Intervention Group (IG) No intervention Group (NIG) Standard state curriculum (OE) Standard state curriculum + 5 min naloxone training (OEN)

Lead Author (Country)	Study Design (type, learners, size, intervention, duration)	Outcome Measures	Key Findings
Williams [25](England)	Randomized controlled trial; non-blinded Family members of people who use heroin N = 187; 123 completed Opioid Education & Naloxone (OEND) training Facilitator led group education, skill practice, 60 minutes (n=69) Passive pamphlet (n=54 control)	3-month follow up Experiment: 79% retention Control: 72% retention Self-completion/reported Primary outcome measures: Opioid Overdose Knowledge Scale (OOKS; range 0-45) Opioid Overdose Attitudes Scale (OOAS; range 28-140)	13 (11%) participants witnessed an overdose @ 3 months: Naloxone use in 8 instances: 3x control group and 5x facilitator-trained group. 2 facilitator trained administered naloxone; in other instances, naloxone was given by ambulance personnel. At follow up significant increase in knowledge for facilitator led training At follow up significant increase in attitude for facilitator led & passive training
Dunn [19] (United States)	Randomized pre-post trial; non-blinded People undergoing outpatient opioid detoxification N=76; Opioid overdose information Pamphlet (N = 25) Computer (N = 24) Computer + Mastery (N = 27) Pre, post testing	Post, 1-month, 3-month follow up 43 completed 1 or 3-month follow up; 57 % retention Self-completion/reported Primary outcome measures were changes from pre- to post-intervention in knowledge of opioid effects, opioid overdose symptoms, and recommended opioid overdose responses.	@ post intervention significant increase in opioid knowledge by computer groups v. pamphlet @ post intervention significant increase by all groups in opioid response knowledge (41.8% to 73.8%) No difference in groups’ opioid overdose knowledge pre or post, as it was initially high, and assumed to be a group characteristic based on personal experience. By the one (81%) and three (77%) month follow-up visits, most participants in the completed sample provided a urine sample that tested positive for an opioid, indicating relapse to opioid use.
Espelt [21] (Spain)	Quasi-experimental, pre-implementation to post-systematic implementation overdose prevention training People who inject opioids Time before and after a standardized education program established Pre-Intervention Group Without Training in Overdose Prevention (PREIGW, n=529) Pre-Intervention Group with Sporadic Training in Overdose Prevention (PREIGS, n=196) Comparison Group (CG, n=502) Intervention Group (IG, n=220) Pre-intervention 2008-2009; Systematic-intervention 2010-2011	12-month follow-up Before (n = 725) and after (n = 722); 99% retention Primary outcome measure was knowledge of overdose prevention	Knowledge of overdose prevention increased after implementing systematic training program. Compared to the PREIGW, the IG gave more correct answers (IRR = 1.40;95%CI:1.33– 1.47), and fewer incorrect answers (IRR = 0.33;95%CI:0.25–0.44). IG: 158 (72%) received naloxone, of whom 94 (59%) reported having witnessed ≥1 overdose in the 12 months prior to the interview, 68% of whom (n = 64) helped the sufferer (59% of these administered naloxone), thus 40% used the kit in response to an overdose they witnessed. Knowledge about overdose prevention was greater after the implementation of systematic program; Incidence Rate Ratio of correct answers 1.09 (CI 1.04-1.16) PREIGS and 1.40 (CI 1.33-1.47) IG
Franko [22] (United States)	Randomized to overdose response training College students Overdose identification and intervention Standard web presentation of overdose recognition and response (control, n=64) Enhanced web presentation (voice over .ppt, video of overdose simulation; n=69)	Participants’ actions evaluated following training Primary outcome measure was behavior No follow-up listed	2 min to complete enhanced versus 2:10 min for standardized Simulation response differences (significant frequency differences in favor of enhanced web presentation) Determines if the patient has a pulse (checks pulse) Determines if the patient is breathing (e.g., chest rise/fall, put ear to nose) Slightly tilts patient’s head to expose nasal passage better Properly administers naloxone
Dwyer [20] (United States)	Cross-sectional Emergency department patients seen by licensed drug counselors N=415 Overdose education and response Standard state curriculum (OE, n=359) Standard state curriculum + 5 min naloxone training (OEN, n=56)	Median time between ED index visit and survey completion was 12 months for OE only (range: 8-17 months), and 11 months for OEN (range: 5-19 months) 12% retention rate Self-completion/reported Primary outcome measure was knowledge	Those responding to an overdose (27) 14 of 19 OEN called 9-1-1 versus 3 of 8 in the OE group (non-sig) No sig difference in rescue breathing rates 6 of 19 OEN administered nasal naloxone versus 0 of 8 OE Only knowledge difference was OEN recognizing that periods of opioid abstinence impacted chances of overdose
Jones [23] (United States)	Quasi-experimental, pre-post test People who use heroin Overdose training mandated by the New York State Department of Health (NYSDOH) N=84 Experimental: pre, posttest (n=44) Control: pretest only (n = 40) Training period 15 minutes	Experimental group completed the questionnaire immediately prior to and following training. Self-completed/reported Primary outcome measure was knowledge	Post intervention confidence in naloxone use was significantly higher (9.4) in comparison to the untrained group [t(82) = 16.17, p < 0.05], and their pre-training baseline [t(43) = 22.09, p < 0.05].
Doe-Simkins [18] (United States)	Retrospective cohort study People who use substances (participants) Opioid Overdose Training N = 4,926 Rescues self-reported (pre-training n=91; post-training n=508 Survey reporting period 2006-2010	Self-completed/reported Primary outcome measure was behavior No follow-up listed	No statistically significant differences in help-seeking (call for help/ 9-1-1 rescue breathing staying with the poisoned victim success of naloxone administration No sig difference in drug usage among participants
Lott [24] (United States)	Quasi-experimental, pre-post Outpatient treatment for people with Opioid Use Disorder Embedded 30-45 Opioid Overdose Prevention within a 4-week program – Small group lecture Intervention group (IG; n=43); follow-up (n=16) No intervention Group (NIG; n=14); follow-up (n=6)	3-month follow-up IG: 37% retention NIG: 43% retention Primary outcome measure was Opioid Overdose Knowledge Scale (OOKS)	IG showed greater improvement in the Opioid Overdose Knowledge Scale (OOKS) total score and the Naloxone Use subdomain score in comparison to the control group. Post-hoc comparisons of the IG versus NIG follow-up scores for OOKS Total and Naloxone use were not significant. IG pre-to follow-up; no change in naloxone access reported following this educational intervention IG: no reported use of naloxone in past year at follow-up

From self-reports of those helping, first aid interventions (CPR or rescue breathing, naloxone administration) were identified in two comparison studies (n=173) and showed no statistical difference between those trained and not trained regarding helping in an opioid overdose poisoning [18,20]. Similarly, PWIO family members trained in and provided Take Home Naloxone (THN) responded the same as the control group [25]. In a simulation study, Franko et al. (2019) found a brief enhanced web education intervention, when compared to a standard web presentation (<3 minutes), increased frequency of pulse check, breathing check (e.g., chest rise/fall, put ear to nose), tilting head to assist breathing, and properly administered naloxone. 

A variety of assessment tools used resulted in reporting of heterogeneous educational outcomes between the comparison studies, which made it difficult to compare outcomes between studies. Outcomes included: knowledge of opioid overdose risks, identification of opioid overdose, knowledge of opioid overdose response, knowledge of opioid antagonist (naloxone), skill to provide naloxone, attitude/willingness to aid, and attitude to call EMS and/or involve law enforcement. The 59 studies provided a scoping perspective on the main populations being trained and relative outcomes being sought by training organizations. The results demonstrate that lay responders are engaged and able to learn early recognition, first aid, and involve advanced care appropriately.

Discussion

The continuum of first aid education spans multiple domains of public health, one of which is the knowledge and skill of first aid. Each study addressed the knowledge and or skills used in an opioid overdose poisoning situation. Depending on the goals of the training organization, or in some cases the researcher, attention to attitude and prevention were prioritized, while others focused on the administration of naloxone. An optimal balance between the domains of first aid education was not apparent or inequities rationalized, which leaves readers to speculate as to the design of the intervention. The Chain of Survival Behavior illustrates that when a link is not addressed the chain is broken, which decreases survival. In these cases, programs that emphasize skills need some evidence or a system to provide access to the medication itself. In this review to a varying degree, when naloxone was offered at the end of training, people used it, compared to those who didn’t receive it post-education [20,21]. There is no clear evidence that the education itself improved odds of receiving naloxone; however, the influential variable may be the ease of access to the medication post-education.

The EIT taskforce identified and raised the following as limitations and possibilities within opioid overdose education. Overall, the inconsistent reporting of educational interventions makes comparison between studies challenging. The use of the Guideline for Reporting Evidence-based practice Educational interventions and Teaching (GREET) checklist for educational interventions would help standardize future analysis [26]. Additionally, with only one RCT and seven other studies with control groups, a lack of experimental rigor limits comparison and strength of any future recommendations. Likewise, a prospective means to validate self-reported use of first aid/naloxone in these emergencies should be developed to have higher confidence in the outcomes. For example, if emergency services responded, they could corroborate potential overdoses, naloxone administration, and clinical outcomes to increase validity through triangulation. This could be complicated as debate exists regarding the need for hospitalization post-overdose reversal.

Simulation studies may offer an indirect means to observe learned behaviors for emergency response. Franko, Distefano, and Lewis [22] used simulation to test differences between trained and non-trained college students. Unique to this study was the injection of a stressor, a panicked bystander. Kobayashi et al. [27] educated people in prison and then tested a cohort one month after release in a simulated environment that had distractor decoys, common to overdose treatment errors. Future work on standardizing simulations would help assessment and evaluation across studies.

The interventions that reported training people in the skills also reported on clinical outcomes 61% of the time (11/18), compared to no-skill intervention 45% (19/42). As noted above, the positive outcomes for PWIO were also higher in skill inclusive training (91%- 10/11 v.79%- 15/19). Brief training (<15 minutes) of PWIO non-medically without skills, also appears beneficial for those who overdosed; perhaps this is due to personal and social experience with drugs. In addition, standalone education (16-60 minutes) with skill training for PWIO medically and non-medically and first responders appears to show benefits for clinical outcomes. 

In trying to understand the limitations of the current evidence base for OEND, the EIT taskforce identified gaps that, if filled, would strengthen future first aid education guidelines. Validation of an assessment tool(s) that works across populations to report educational outcomes would help with future metanalysis. Specifically, for this would benefit describing opioid knowledge and risks, early recognition of life threats and suspicion of opioid overdose poisoning, first aid for positioning, resuscitation skills, naloxone administration, accessing additional help and knowledge of self-recovery for the poisoned victim and first aider. Within the context of opioid overdoses, future research is needed to explore the knowledge or behaviors that help or hinder a first responder to move between first aid education domains to care for a person. This would broaden the perspective of leverage points. Comparing educational approaches within populations of potential responders (e.g., PWIO, friends and family, teachers, first responders, and unrelated bystanders) and comparison of an educational approach between groups would help with generalizing outcomes. 

To help fill in the gaps, inquiry could be better served from an educational or social behavioral theory/models. Social-ecological relationships between bystander and risk/opportunity to aid could be a grounding model for future inquiry. For example, costs and opportunities for training PWIO non-medically directly versus random bystanders; differences in educational and helping motivations between groups; length of intervention, and personal attributes such as self-efficacy. Practically, identifying evidence to support the timing of naloxone within a resuscitation sequence would help standardize education. Evidence-based practice from clinical sources needs to be extrapolated and validated for lay responders to recognize opioid overdoses, to again help standardize education. 

A more systematic approach could be implemented to discern where the value of any one approach contributes to the most meaningful outcomes. As the clinical evidence for naloxone solidifies, the educational elements and efficiencies are now being considered to position educators and organizations for local implementation, which when viewed together are a formula for survival [11].

## Conclusions

Public health’s core function of policy development charges us to inform, educate, and empower people about the risks in their environment. Work to mobilize community partnerships and actions to identify and address opioid overdose poisoning is evidenced in the 59 studies, along with a diversity of approaches based on a variety of outcomes. However, the educational literature is sparse, of low quality, lacks quality measures and effective methodologies, and suffers from self-reported and highly inconsistent endpoints, making outcome comparisons challenging, if not impossible. Opportunity exists to develop consistent policies and standardized plans that support individual and community efforts through evidence-based guidelines, particularly to the domains of first aid education, so that educators and organizations can meet the needs of their learners.
